# Real-world outcomes of avelumab plus axitinib in patients with advanced renal cell carcinoma in Japan: long-term follow-up from the J-DART2 retrospective study

**DOI:** 10.1007/s10147-024-02618-9

**Published:** 2024-11-16

**Authors:** Taigo Kato, Junya Furukawa, Nobuyuki Hinata, Kosuke Ueda, Isao Hara, Fumiya Hongo, Ryuichi Mizuno, Teppei Okamoto, Hiroshi Okuno, Takayuki Ito, Masahiro Kajita, Mototsugu Oya, Yoshihiko Tomita, Nobuo Shinohara, Masatoshi Eto, Hirotsugu Uemura

**Affiliations:** 1 Department of Urology, Osaka University Graduate School of Medicine, 2-2 Yamadaoka, Suita, Oska 565-0871 Japan; 2 Department of Urology, Kobe University Graduate School of Medicine, 7-5-1 Kusunoki-cho, Chuo-ku, Kobe, 650-0017 Japan; 3 Department of Urology, Graduate School of Biomedical and Health Sciences, Hiroshima University, Kasumi 1-2-3, Minami-ku, Hiroshima, 734-8551 Japan; 4 Department of Urology, Kurume University School of Medicine, Asahi-Machi 67, Kurume-shi, Fukuoka, 830-0011 Japan; 5 Department of Urology, Wakayama Medical University, 811-1 Kimiidera, Wakayama, 641-8509 Japan; 6 Department of Urology, Kyoto Prefectural University of Medicine, 465 Kajii-cho, Kawaramachi-Hirokoji, Kamigyo-ku, Kyoto, 602-8566 Japan; 7 Department of Urology, Keio University School of Medicine, 35 Shinanomachi, Shinjuku-ku, Tokyo, 160-8582 Japan; 8 Department of Urology, Hirosaki University Graduate School of Medicine, 5 Zaifu-cho, Hirosaki, Aomori 036-8562 Japan; 9 Department of Urology, National Hospital Organization Kyoto Medical Center, 1-1 Fukakusa-Mukaihatacho, Fushimiku, Kyoto, 612-8555 Japan; 10 Medical Department, Merck Biopharma Co., Ltd., Tokyo, Japan, an affiliate of Merck KGaA, 1-8-1 Shimomeguro, Meguro-ku, Tokyo, 153-8926 Japan; 11 Department of Urology, Keio University School of Medicine, 35 Shinanomachi, Shinjuku-ku, Tokyo, 160-8582 Japan; 12 Departments of Urology and Molecular Oncology, Niigata University Graduate School of Medical and Dental Sciences, 1-757 Asahimachi Street, Chuo Ward, Niigata, 951-8510 Japan; 13 Department of Renal and Genitourinary Surgery, Graduate School of Medicine, Hokkaido University, Kita15, Nishi7, Kita-Ku, Sapporo, Hokkaido 060-8638 Japan; 14 Department of Urology, Graduate School of Medical Sciences, Kyushu University, 3-1-1 Maidashi, Higashi-ku, Fukuoka, 812-8582 Japan; 15 Department of Urology, Kindai University Faculty of Medicine, 377-2 Ohno-Higashi, Osakasayama, Osaka 589-8511 Japan

**Keywords:** Renal cell carcinoma, Avelumab, Axitinib, Real world, Retrospective, Japan

## Abstract

**Background:**

Avelumab + axitinib was approved for advanced renal cell carcinoma (aRCC) in Japan in December 2019. We report long-term real-world outcomes with first-line avelumab + axitinib from the J-DART2 study in Japan.

**Methods:**

J-DART2 was a multicenter, noninterventional, retrospective study examining clinical data from patients with curatively unresectable locally advanced or metastatic RCC who started treatment with first-line avelumab + axitinib in Japan between December 2019 and October 2022. Endpoints included patient characteristics, treatment patterns, and outcomes.

**Results:**

Data from 150 patients across 19 sites were analyzed; median follow-up was 18.7 months (95% CI, 16.3–20.6 months). Median age was 70.5 years; 26.0% of patients were aged ≤64 years, 42.7% were aged 65–74 years, and 31.3% were aged ≥75 years. International Metastatic RCC Database Consortium risk was favorable in 26.0%, intermediate in 54.7% (1 risk factor in 30.7%; 2 risk factors in 24.0%), and poor in 19.3% of patients. Median progression-free survival (PFS) was 17.1 months, with 1- and 2-year PFS rates of 57.7% and 37.5%, respectively. Median overall survival (OS) was not reached, with 1- and 2-year OS rates of 90.6% and 84.7%, respectively. Objective response rate was 53.3%; disease control rate was 88.9%. Outcomes were similar across age groups, including patients aged ≥75 years.

**Conclusions:**

J-DART2 is the largest retrospective study to report long-term real-world outcomes in patients with aRCC treated with avelumab + axitinib in Japan. Findings were similar to those observed in previous studies and support the benefit of avelumab + axitinib in clinical practice in Japan.

## Introduction

The kidney and other urinary organs were the ninth most common cancer site in Japan in 2023, accounting for 31,500 new cases and 10,100 deaths [1]. Renal cell carcinoma (RCC) accounts for approximately 90% of kidney cancers [2]. Although the incidence of RCC is lower in Japan than in Western countries, it continues to increase annually [3, 4]. Based on results from several clinical trials, international guidelines recommend immune checkpoint inhibitor (ICI)-based regimens as the first-line (1L) standard-of-care treatment for advanced RCC (aRCC), including combination treatment with an ICI and a tyrosine kinase inhibitor (TKI) [2, 5–10].

Combination treatment with avelumab, an anti-PD-L1 monoclonal antibody, and axitinib, a multitargeted TKI that inhibits vascular endothelial growth factor receptors, is approved as a 1L treatment for patients with aRCC in various countries worldwide [11–15]. This approval was based on results from the phase 3 JAVELIN Renal 101 trial (NCT02684006), which demonstrated significantly longer median progression-free survival (PFS) and a higher objective response rate (ORR) with avelumab + axitinib vs. sunitinib, the prior standard of care (median PFS: 13.9 vs. 8.5 months, respectively; hazard ratio [HR], 0.67 [95% confidence interval (CI), 0.568–0.785], *p* < 0.0001; ORR, 59.3% vs. 31.8%) [16–18]. In a subgroup analysis of patients from JAVELIN Renal 101 who were enrolled in Japan (*n* = 67), avelumab + axitinib also showed longer PFS and a higher ORR vs. sunitinib (median PFS: 16.6 vs. 11.2 months, respectively; HR, 0.66 (95% CI, 0.296–1.464); ORR, 60.6% vs. 17.6%) [19]. Based on these findings, avelumab + axitinib was the first anti-PD-L1 ICI + TKI combination treatment approved for patients with aRCC in Japan in December 2019 [15]. The Japanese Urological Association clinical practice guidelines also recommended avelumab + axitinib as 1L treatment for patients with clear cell RCC in 2022 [9].

The incidence of cancer increases as populations age, with >50% of patients with cancer in high-income countries aged >70 years. Japan has a rapidly aging population, with a higher proportion of individuals aged ≥65 years compared with most countries [20]. It has been hypothesized that older patients may benefit less from immunotherapy because of immune senescence—a decline in immune activity that hinders the ability to combat carcinogenesis and promotes cancer development [21, 22]. A subgroup analysis from JAVELIN Renal 101 showed favorable efficacy and consistent tolerability with avelumab + axitinib vs. sunitinib across age groups, including patients aged ≥75 years [23]. Real-world data are needed to assess the effectiveness of 1L avelumab + axitinib in patients with aRCC receiving routine clinical care in Japan, including older patients.

In the real-world J-DART study (NCT05012865), a clinically meaningful benefit was observed in patients (*N* = 48) with aRCC treated with 1L avelumab + axitinib 1 year after its approval in Japan, including patients aged <75 years or ≥75 years. While J-DART contributed to the understanding of real-world treatment patterns of avelumab + axitinib soon after its approval, the study was limited by small patient numbers [24]. In addition, a post-marketing surveillance (PMS) study in Japan confirmed the acceptable safety and tolerability of avelumab + axitinib in patients with aRCC treated in clinical practice [25]. Here we report findings from the larger observational J-DART2 study (*N* = 150), which examined long-term, real-world baseline characteristics and treatment outcomes in patients with aRCC treated with 1L avelumab + axitinib and followed for ≥2 years in Japan. In addition, we report results from subgroup analyses in patients aged ≤64 years, 65–74 years, or ≥75 years.

## Patients and methods

### Study design

J-DART2 (NCT05650164) was a multicenter, observational, retrospective study performed at 19 sites in Japan. Clinical data from patients with aRCC who started treatment with 1L avelumab + axitinib between 20 December 2019 (approval date) and 17 October 2022 were analyzed. The observation period was from the date of the first prescription until 31 October 2022. Data were collected from patient medical records within the follow-up period. All decisions regarding the treatment and clinical management of patients were made by the investigator as part of standard clinical care in a real-world setting and irrespective of the patient’s participation in the study. Ethical review boards at all study sites approved the study protocol and related documentation. The study conduct complied with the Declaration of Helsinki and applicable local laws in Japan.

### Patients

Patients were aged ≥18 years and received 1L avelumab + axitinib for curatively unresectable locally advanced or metastatic RCC (based on the General Rule for Clinical and Pathological Studies on Renal Cell Carcinoma [5th edition]). For surviving patients who had routine visits to the study site, a signed informed consent document was obtained. For surviving patients who had been transferred to another hospital, evidence of oral informed consent was obtained. Deceased patients who met the inclusion criteria were included unless the patient’s family opted out. Patients were ineligible if they were participating in a prospective interventional clinical trial during the follow-up period.

### Objectives and assessments

The primary objective was to describe the demographic and clinical characteristics of patients with aRCC treated with 1L avelumab + axitinib in clinical practice in Japan. The secondary objective was to determine real-world treatment outcomes as measured by endpoints that included ORR and PFS per investigator assessment, overall survival (OS), best overall response, time to treatment discontinuation (TTD; defined as the time from start to end of 1L treatment with avelumab + axitinib for any cause except treatment effectiveness), treatment patterns, use of corticosteroids for immune-related adverse events (irAEs), and subsequent treatment patterns. We report the results of analyses in the overall population and in patients aged ≤64, 65–74, and ≥75 years.

### Statistical analysis

The full analysis population included all enrolled patients at each site during the study period. Effectiveness was assessed in all patients from the full analysis population whose index date was prior to 30 April 2022 to ensure a 6-month follow-up period. Continuous variables were summarized using descriptive statistics. The duration of follow-up was calculated using the reverse Kaplan–Meier method (reversing censoring and event indicators). Qualitative variables were summarized as frequencies and percentages. Time-to-event endpoints (PFS, OS, TTD) were estimated using the Kaplan–Meier method, and corresponding CIs were calculated using the Brookmeyer-Crowley method. Statistical analyses were performed using SAS 9.4 (SAS Institute, Inc.).

## Results

### Patients and treatment

At data cutoff (31 October 2022), 150 patients from 19 sites were included in the effectiveness analysis population. The median observation period was 17.0 months (range, 0.5–32.7 months). Patient baseline characteristics are shown in Table 1. At baseline, the median age was 70.5 years (range, 33–87 years); 39 patients (26.0%) were aged ≤64 years, 64 (42.7%) were aged 65–74 years, and 47 (31.3%) were aged ≥75 years. Most patients were male (*n* = 110 [73.3%]) and had clear cell RCC (*n* = 134 [89.3%]); tumors had a sarcomatoid component in 10 patients (6.7%). The Eastern Cooperative Oncology Group performance status (ECOG PS) was 0 in 116 (77.3%), 1 in 23 (15.3%), and ≥2 in 10 (6.7%) patients. The International Metastatic RCC Database Consortium (IMDC) risk classification was favorable in 39 (26.0%), intermediate with 1 risk factor in 46 (30.7%), intermediate with 2 risk factors in 36 (24.0%), and poor in 29 (19.3%) patients.

**Table 1:** Baseline characteristics in the overall population and in subgroups defined by age

	Overall population (*N* = 150)	≤64 years (*n* = 39)	65–74 years (*n* = 64)	≥75 years (*n* = 47)
Sex, *n* (%)				
Male	110 (73.3)	31 (79.5)	45 (70.3)	34 (72.3)
Female	40 (26.7)	8 (20.5)	19 (29.7)	13 (27.7)
Age, median (range), years	70.5 (33–87)	59 (33–64)	70 (65–74)	78 (75–87)
BMI, *n* (%)				
<25 kg/m^2^	110 (73.3)	25 (64.1)	49 (76.6)	36 (76.6)
≥25 kg/m^2^	39 (26.0)	14 (35.9)	14 (21.9)	11 (23.4)
ECOG PS, *n* (%)				
0	116 (77.3)	37 (94.9)	48 (75.0)	31 (66.0)
1	23 (15.3)	1 (2.6)	9 (14.1)	13 (27.7)
≥2	10 (6.7)	1 (2.6)	7 (10.9)	2 (4.3)
CRP, *n* (%)				
<10 mg/L	104 (69.3)	30 (76.9)	44 (68.8)	30 (63.8)
≥10 mg/L	43 (28.7)	9 (23.1)	19 (29.7)	15 (31.9)
eGFR, *n* (%)				
<60 mL/min	109 (72.7)	27 (69.2)	45 (70.3)	37 (78.7)
≥60 mL/min	40 (26.7)	12 (30.8)	18 (28.1)	10 (21.3)
Pathological classification, *n* (%)				
Clear cell	134 (89.3)	34 (87.2)	56 (87.5)	44 (93.6)
Non-clear cell	10 (6.7)	2 (5.1)	4 (6.3)	0
Unknown	6 (4.0)	3 (7.7)	4 (6.3)	3 (6.4)
Sarcomatoid, *n* (%)	10 (6.7)	3 (7.7)	1 (1.6)	6 (12.8)
No. of metastatic organs, *n* (%)				
0	9 (6.0)	2 (5.1)	6 (9.4)	1 (2.1)
1	73 (48.7)	20 (51.3)	32 (50.0)	21 (44.7)
≥2	68 (45.3)	17 (43.6)	26 (40.6)	25 (53.2)
Nephrectomy, *n* (%)	114 (76.0)	33 (84.6)	48 (75.0)	33 (70.2)
Clinically important comorbidities, *n* (%)	101 (67.3)	19 (48.7)	46 (71.9)	36 (76.6)
IMDC risk group, *n* (%)				
Favorable	39 (26.0)	14 (35.9)	19 (29.7)	6 (12.8)
Intermediate (1 risk)	46 (30.7)	9 (23.1)	18 (28.1)	19 (40.4)
Intermediate (2 risks)	36 (24.0)	9 (23.1)	13 (20.3)	14 (29.8)
Poor	29 (19.3)	7 (17.9)	14 (21.9)	8 (17.0)

Patients with missing information in each category are not shown

*BMI* body mass index, *CRP* C-reactive protein, *ECOG PS* Eastern Cooperative Oncology Group performance status, *eGFR* estimated glomerular filtration rate, *IMDC* International mRCC Database Consortium, *mRCC* metastatic renal cell carcinoma

Compared with patients aged ≤64 years or 65–74 years, a lower proportion of patients aged ≥75 years had an ECOG PS of 0 (94.9% or 75.0% vs. 66.0%, respectively) and a higher proportion had an ECOG PS of 1 (2.6% or 14.1% vs. 27.7%, respectively) (Table 1). In addition, compared with patients aged ≤64 years or 65–74 years, a lower proportion of patients aged ≥75 years had favorable IMDC risk classification (35.9% or 29.7% vs. 12.8%, respectively), a higher proportion had intermediate IMDC with 1 risk factor (23.1% or 28.1% vs. 40.4%, respectively), and a higher proportion had RCC with sarcomatoid features (7.7% or 1.6% vs. 12.8%, respectively).

The median duration of avelumab + axitinib treatment was 10.7 months (interquartile range [IQR], 6.6–18.3 months), the median duration of avelumab treatment was 10.1 months (IQR, 6.0–17.0 months), and the median duration of axitinib treatment was 9.6 months (IQR, 4.9–16.0 months) (Table 2). The median relative dose intensity for avelumab was 100.0% in the overall population and in all age groups and for axitinib was 75.0% in the overall population and 80.0%, 70.0%, and 70.0% in patients aged ≤64 years, 65–74 years, and ≥75 years, respectively (Table 2).

**Table 2:** Treatment exposure for avelumab and axitinib in the overall population and in subgroups defined by age

	Overall population (*N* = 150)	≤64 years (*n* = 39)	65–74 years (*n* = 64)	≥75 years (*n* = 47)
Duration of avelumab + axitinib treatment, median (IQR), months	10.7 (6.6–18.3)	11.5 (8.0–19.4)	10.8 (6.7–19.0)	10.3 (5.0–15.6)
Duration of avelumab treatment, median (IQR), months	10.1 (6.0–17.0)	10.8 (7.2–18.4)	9.9 (6.5–17.9)	9.0 (4.7–14.0)
Duration of axitinib treatment, median (IQR), months	9.6 (4.9–16.0)	10.1 (6.2–16.6)	10.3 (5.8–18.0)	8.3 (3.8–14.6)
Avelumab dose, median (IQR)				
Dose intensity, mg/kg/administration	10.0 (8.2–10.0)	10.0 (8.4–10.0)	10.0 (8.3–10.0)	10.0 (7.9–10.0)
Relative dose intensity, %	100.0 (82.3–100.0)	100.0 (84.0–100.0)	100.0 (82.8–100.3)	100.0 (78.5–100.0)
Axitinib dose, median (IQR)				
Dose intensity, mg/kg/administration	5.2 (4.0–7.2)	6.7 (4.4–9.6)	5.6 (4.2–6.8)	4.6 (3.6–6.7)
Relative dose intensity, %	75.0 (60.0–100.0)	80.0 (70.0–100.0)	70.0 (60.0–100.0)	70.0 (50.0–100.0)

### Clinical outcomes

**Fig. 1:**
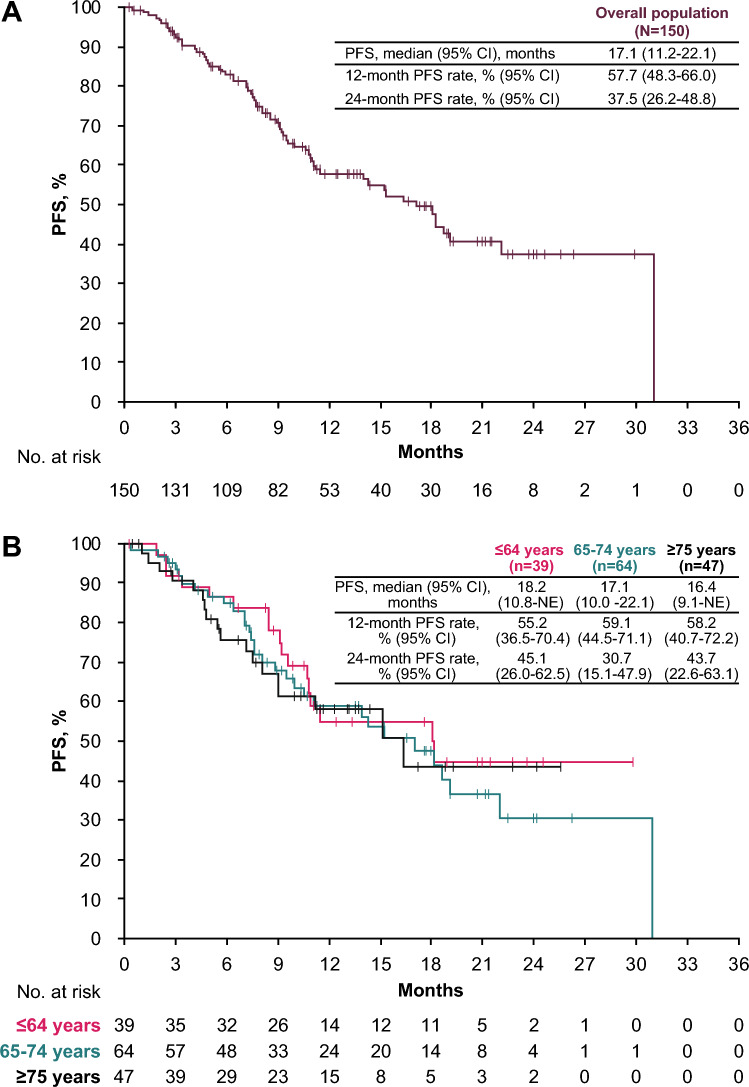
Real-world PFS in **A** the overall population and **B** subgroups defined by age. *CI* confidence interval, *NE* not estimable, *PFS* progression-free survival

Median follow-up for PFS was 13.8 months (95% CI, 12.5–18.0 months). The median PFS in the overall population was 17.1 months (95% CI, 11.2–22.1 months), with 1- and 2-year PFS rates of 57.7% and 37.5%, respectively (Fig. 1A). In patients aged ≤64, 65–74, or ≥75 years, the median PFS was 18.2 months (95% CI, 10.8 months-not estimable [NE]), 17.1 months (95% CI, 10.0–22.1 months), and 16.4 months (95% CI, 9.1 months-NE); the 2-year PFS rates were 45.1%, 30.7%, and 43.7%, respectively (Fig. 1B).

**Fig. 2:**
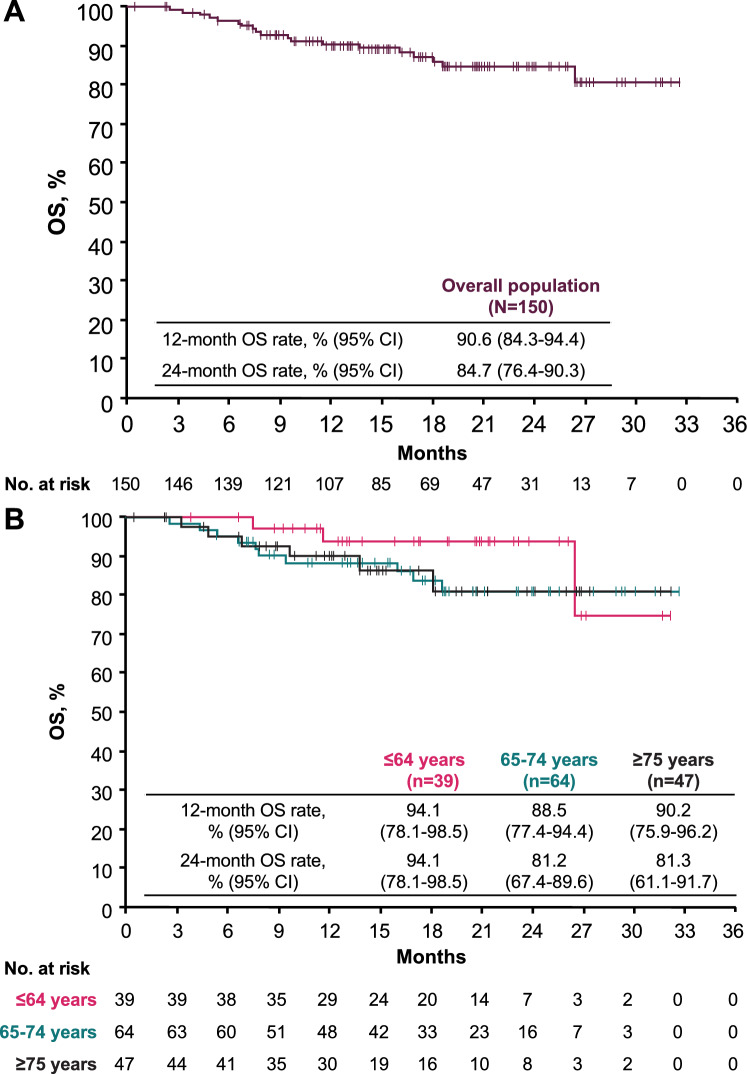
Real-world OS in **A** the overall population and **B** subgroups defined by age.* CI* confidence interval, *OS* overall survival

**Fig. 3:**
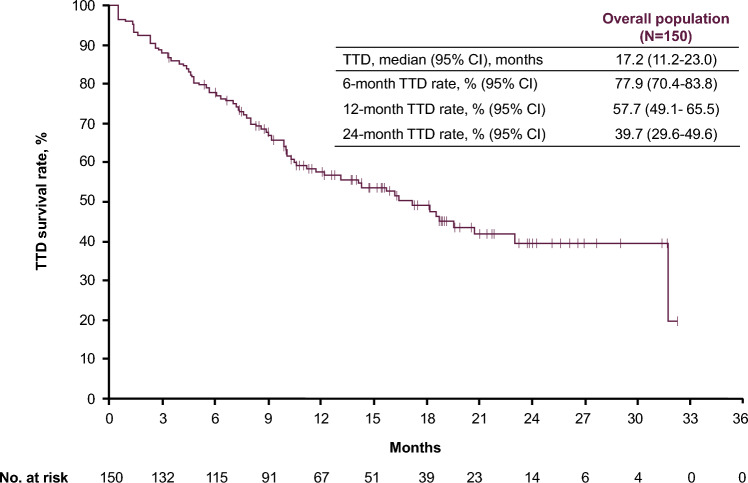
Real-world TTD survival rate in the overall population. *CI* confidence interval, *TTD* time to treatment discontinuation

Median follow-up for OS was 18.7 months (95% CI, 16.3–20.6 months). The median OS was not reached in the overall population or in any of the subgroups defined by age (Fig. 2). In the overall population, 1- and 2-year OS rates were 90.6% and 84.7%, respectively (Fig. 2A). In patients aged ≤64, 65–74, or ≥75 years, 2-year OS rates were 94.1%, 81.2%, and 81.3%, respectively (Fig. 2B). The median TTD for avelumab + axitinib was 17.2 months (95% CI, 11.2–23.0 months), with 6-month, 1-year, and 2-year TTD rates of 77.9%, 57.7%, and 39.7%, respectively (Fig. 3).

Among 135 patients assessed for best overall response, the ORR was 53.3% (95% CI, 44.6–62.0%) and the disease control rate (DCR) was 88.9% (95% CI, 82.3–93.6%) (Table 3). In patients aged ≤64, 65–74, or ≥75 years, ORRs were 55.3% (95% CI, 37.2–69.9%), 49.1% (95% CI, 29.9–55.2%), and 57.1% (95% CI, 41.0–72.3%) and DCRs were 84.2% (95% CI, 66.5–92.5%), 87.3% (95% CI, 62.8–85.0%), and 95.2% (95% CI, 83.8–99.4%), respectively.

**Table 3:** Objective response in the overall population and in subgroups defined by age

	Overall population (*n* = 135)^a^	≤64 years (*n* = 38)	65–74 years (*n* = 55)	≥75 years (*n* = 42)
BOR, *n* (%)				
Complete response	12 (8.9)	4 (10.5)	6 (10.9)	2 (4.8)
Partial response	60 (44.4)	17 (44.7)	21 (38.2)	22 (52.4)
Stable disease	48 (35.6)	11 (28.9)	21 (38.2)	16 (38.1)
Progressive disease	14 (10.4)	6 (15.8)	6 (10.9)	2 (4.8)
Not evaluable	1 (0.7)	0	1 (1.8)	0
ORR, *n* (%) [95% CI]	72 (53.3) [44.6–62.0]	21 (55.3) [37.2–69.9]	27 (49.1) [29.9–55.2]	24 (57.1) [41.0–72.3]
DCR, *n* (%) [95% CI]	120 (88.9) [82.3–93.6]	32 (84.2) [66.5–92.5]	48 (87.3) [62.8–85.0]	40 (95.2) [83.8–99.4]

*BOR* best overall response, *CI* confidence internal, *DCR* disease control rate, *ORR* objective response rate

^a^Best overall response not reported in 15 patients (1 patient aged ≤64 years, 9 patients aged 65–74 years, and 5 patients aged ≥75 years)

### Treatment discontinuations and subsequent treatment

At the end of the follow-up period, 75 patients (50.0%) were still receiving avelumab + axitinib treatment, including 22 patients (56.4%) aged ≤64 years, 30 (46.9%) aged 65–74 years, and 23 (48.9%) aged ≥75 years (Table 4). In patients who discontinued avelumab + axitinib (*n* = 75 [50.0%]), the most common reason for discontinuation was progressive disease (*n* = 37 [24.7%]), followed by occurrence of adverse events (*n* = 31 [20.7%]). In 54 patients (36.0%) who received a subsequent treatment, cabozantinib was most frequently administered followed by nivolumab (Table 4). Of patients who discontinued avelumab + axitinib treatment due to disease progression (*n* = 37) or adverse events (*n* = 31), 30 (81.1%) and 16 (51.6%) received a second-line treatment, respectively. Median time from avelumab + axitinib discontinuation to second-line treatment was 0.6 months (range, 0.03–5.6 months) in the overall population, 0.5 months (range, 0.03–2.8 months) in patients who discontinued due to disease progression, and 1.3 months (range, 0.1–5.6 months) in patients who discontinued due to adverse events. Rates of treatment discontinuation, reasons for treatment discontinuation, and subsequent treatments were generally similar across age groups.

**Table 4:** Treatment discontinuation and subsequent treatments in the overall population and in subgroups defined by age

	Overall population (*N* = 150)	≤64 years (*n* = 39)	65–74 years (*n* = 64)	≥75 years (*n* = 47)
Ongoing treatment at data cutoff, *n* (%)	75 (50.0)	22 (56.4)	30 (46.9)	23 (48.9)
Discontinued treatment, *n* (%)	75 (50.0)	17 (43.6)	34 (53.1)	24 (51.1)
Reasons for treatment discontinuation, *n* (%)^a^				
Progressive disease	37 (24.7)	9 (23.1)	18 (28.1)	10 (21.3)
Adverse event	31 (20.7)	6 (15.4)	15 (23.4)	10 (21.3)
Other	12 (8.0)	4 (10.3)	4 (6.3)	4 (8.5)
Received subsequent treatment, *n* (%)	54 (36.0)	16 (41.0)	21 (32.8)	17 (36.2)
Treatment regimen, *n* (%)				
Cabozantinib monotherapy	38 (25.3)	12 (30.8)	16 (25.0)	10 (21.3)
Nivolumab monotherapy	9 (6.0)	1 (2.6)	3 (4.7)	5 (10.6)
Nivolumab + cabozantinib	2 (1.3)	2 (5.1)	0	0
Axitinib monotherapy	2 (1.3)	0	1 (1.6)	1 (2.1)
Pazopanib monotherapy	2 (1.3)	0	1 (1.6)	1 (2.1)
Everolimus monotherapy	1 (0.7)	1 (2.6)	0	0

^a^Patients with >1 reason for discontinuation are included in all relevant rows

### Treatment for irAEs

Twenty-two patients (14.7%) received corticosteroid treatment at any dose for irAEs for a median of 2.7 months (range, 0.03–19.5 months) and 11 (7.3%) received high-dose corticosteroid treatment for a median of 2.8 months (range, 0.03–19.5 months) (Table 5). In patients aged ≤64 years, 65–74 years, or ≥75 years, 9 (23.1%), 5 (7.8%), and 8 (17.0%) patients received corticosteroid treatment at any dose and 5 (12.8%), 2 (3.1%), and 4 (8.5%) received high-dose corticosteroid treatment, respectively. The duration of corticosteroid treatment is shown in Table 5.

**Table 5:** Use of corticosteroid for irAEs

	Overall population (*N*= 150)	≤64 years (*n* = 39)	65–74 years (*n* = 64)	≥75 years (*n* = 47)
Corticosteroids, *n* (%)	22 (14.7)	9 (23.1)	5 (7.8)	8 (17.0)
Duration of treatment, median (range), months	2.7 (0.03–19.5)	2.1 (0.03–19.5)	5.5 (0.1–16.1)	1.5 (0.03–17.0)
High-dose corticosteroids, *n* (%)^a^	11 (7.3)	5 (12.8)	2 (3.1)	4 (8.5)
Duration of treatment, median (range), months^b^	2.8 (0.03–19.5)	2.8 (0.03–19.5)	10.8 (5.5–16.1)	0.1 (0.03–2.8)

*irAE* immune-related adverse event

^a^High dose was defined as prednisolone-equivalent corticosteroid doses of ≥40 mg

^b^Duration period for ≥1 dose of high-dose corticosteroid

## Discussion

J-DART2 represents the largest retrospective, observational study to provide real-world data on long-term outcomes in patients with aRCC treated with 1L avelumab + axitinib in Japan. Patient outcomes in the overall population of J-DART2 are consistent with those observed in the pivotal JAVELIN Renal 101 clinical trial (including subgroup analyses of patients enrolled in Japan and older patients), the smaller J-DART real-world study, and the PMS [16, 23–25].

Compared with the avelumab + axitinib arm of the JAVELIN Renal 101 trial, the patient population in J-DART2 had a higher proportion of older patients and patients who would not have been eligible for the clinical trial, including those with an ECOG PS ≥2 or non-clear cell RCC [16, 23]. Baseline characteristics in J-DART2 were generally consistent with those reported in J-DART [24] and PMS analyses [25].

The median PFS with 1L avelumab + axitinib in J-DART2 (17.1 months [95% CI, 11.2–22.1 months]) was similar to that reported in JAVELIN Renal 101, including the overall avelumab + axitinib arm and Japanese subgroup analyses (13.9 months [95% CI, 11.1–16.6 months] and 16.6 months [95% CI, 8.1 months-NE], respectively) and in J-DART (15.3 months [95% CI, 9.7 months-NE]) [18, 19, 24]. The ORR in J-DART2 (53.3%) was also similar to that in JAVELIN Renal 101, including the overall avelumab + axitinib arm and Japanese subgroup analysis (59.3% and 60.6%, respectively) and J-DART (48.8%) [18, 19, 24]. While OS was not reached by the data cutoff in J-DART2, the 12-month OS rate (90.6%) was similar to that in JAVELIN Renal 101 (86%) [26]. Although OS analyses did not reach statistical significance at the final analysis of JAVELIN Renal 101, results for PFS and ORR consistently favored avelumab + axitinib and support outcomes from the J-DART2 study in clinical practice in Japan [27].

Recent data from clinical trials and meta-analyses have suggested that older patients with aRCC can still benefit from ICI-based regimens [21–23, 28, 29]. A comprehensive review of ICI-based treatments in older patients with aRCC concluded that the available data do not suggest a lower efficacy compared with younger patients [21]. Because Japan has a rapidly aging population, J-DART2 examined long-term clinical outcomes in older patients with aRCC treated with 1L avelumab + axitinib. Compared with the avelumab + axitinib arm from the JAVELIN Renal 101 trial, the study population in J-DART2 had a higher proportion of older patients (aged ≤64 years, 61.3% vs. 26.0%; aged 65–74 years, 31.2% vs. 42.7%; aged ≥75 years, 7.5% vs. 31.3%, respectively) [23]. Baseline characteristics in J-DART2 were generally balanced across age groups, but the subgroup of patients aged ≥75 years had a higher proportion of patients with an ECOG PS of 1, tumors with sarcomatoid features, or intermediate IMDC risk classification (1 risk factor). PFS, OS, ORR and DCR were consistent across age groups in J-DART2, supporting the benefit of avelumab + axitinib treatment in patients with aRCC, including those aged ≥75 years.

In J-DART2, 7.3% of patients received high-dose corticosteroid treatment for irAEs, which is lower than in the JAVELIN Renal 101 trial (14.5%) but similar to that in subgroup analyses in Japan (9.1%) and in J-DART (6.3%) [18, 19, 24]. Rates of high-dose corticosteroid administration for irAEs were generally low across age subgroups, suggesting that avelumab + axitinib treatment has a manageable safety profile regardless of age.

Half of the patients enrolled in J-DART2 were still receiving 1L avelumab + axitinib at the end of the follow-up period. Of patients who discontinued treatment, 36% received a subsequent anticancer treatment, which is consistent with the JAVELIN Renal 101 trial (46.2%) [18]. Median time from avelumab + axitinib discontinuation to second-line treatment was longer in patients who discontinued due to adverse events vs. those who discontinued due to disease progression (1.3 vs. 0.5 months). However, >50% of patients who discontinued treatment due to adverse events received a second-line treatment, suggesting that appropriate management of adverse events may help the implementation of sequential treatment. Overall, the rates of treatment discontinuation and subsequent anticancer treatments were generally consistent between patients aged ≤64 years, 65–74 years, and ≥75 years.

Our study had some limitations. As a retrospective study, only existing data reported in patient records was available for analysis, and missing data may have affected the accuracy of estimations. The evaluation of disease response may have differed at each site, which might also have led to variations in estimated values. In addition, high-volume centers were preferentially selected for this study, which could have led to site selection and outcome reporting biases. Therefore, the study results may not accurately reflect clinical outcomes for all patients with aRCC in Japan. In addition, the patient population and methods of assessment in this study were different from those in the JAVELIN Renal 101 trial; thus, comparisons must be interpreted with caution. Lasty, J-DART2 did not collect data on adverse events to avoid overlap with a PMS study that has analyzed adverse events data for avelumab + axitinib in Japan [25].

## Conclusion

J-DART2 provides real-world data on the long-term effectiveness of 1L avelumab + axitinib in patients with aRCC in clinical practice in Japan, including older patients. Avelumab + axitinib was associated with clinically meaningful benefits across patient age groups, and outcomes were generally consistent with those reported previously. J-DART2 further supports the continued use of avelumab + axitinib as a 1L standard of care for patients with aRCC.

## Data Availability

Any requests for data by qualified scientific and medical researchers for legitimate research purposes will be subject to Merck’s Data Sharing Policy. All requests should be submitted in writing to Merck’s data sharing portal (https://www.merckgroup.com/en/research/our-approach-to-research-and-development/healthcare/clinical-trials/commitment-responsible-data-sharing.html). When Merck has a co-research, co-development, or co-marketing or co-promotion agreement, or when the product has been out-licensed, the responsibility for disclosure might be dependent on the agreement between parties. Under these circumstances, Merck will endeavor to gain agreement to share data in response to requests.
